# Peer Review for “Impact of a Point-of-Care Ultrasound Training Program on the Management of Patients With Acute Respiratory or Circulatory Failure by In-Training Emergency Department Residents (IMPULSE): Before-and-After Implementation Study”

**DOI:** 10.2196/72144

**Published:** 2025-03-03

**Authors:** Anonymous

**Keywords:** point-of-care ultrasonography, training program, acute respiratory failure, acute circulatory failure, emergency department

*This is the peer-review report for “Impact of a Point-of-Care Ultrasound Training Program on the Management of Patients With Acute Respiratory or Circulatory Failure by In-Training Emergency Department Residents (IMPULSE): Before-and-After Implementation Study.”*

## Round 1 Review

### General Comments

This paper [1] researches an essential component of point-of care ultrasonography. As this modality is rapidly evolving, evaluation of the impact on patient management and outcomes as well as cost-effectiveness is essential. Both subjects discussed in the paper result in a highly relevant manuscript. Even though the authors discuss relevant subjects, there are some problems with the manuscript.

### Specific Comments

#### Major Comments

1. The title of the manuscript suggests that the authors researched the impact of ultrasound after implementation. However, as stated in the Methods section, ultrasound is already used by senior physicians. Thus, the impact of ultrasound after implementation is not researched but rather the impact of ultrasound used by residents. I suggest that the authors clarify that this is a feasibility and impact study on the implementation of point-of-care ultrasound (POCUS) used by residents in the emergency department (ED) in the title and Abstract.

2. The authors state that patients were not included consecutively due to logistics in phase 2. This results in a high risk of bias in the included patients. Please include in the CONSORT (Consolidated Standards of Reporting Trials) diagram the number of patients that were eligible and were excluded based on exclusion criteria or missed.

3. It is unclear how many residents were performing the ultrasound examinations included in the analysis: the Methods section state that there was only 1 resident at the ED in both phases, while in the Results section, it states that there were 12 residents trained. Please clarify.

4. The authors state that they chose a before-and-after implementation to evaluate the effect of POCUS to avoid contamination. However, before the implementation, POCUS was already used by senior physicians, which raises the question if POCUS was indeed not used in phase 1 of the trial.

5. Interestingly, in the Discussion section, the author discussed that the publication of Msolli et al [2] did not find an improvement of diagnostic accuracy. It would be interesting to discuss why this is the case.

6. In the Discussion and Conclusion, it is suggested that the use of POCUS might lead to a decrease in hospital mortality. Since this is an observational study in which, just as the authors state, a diagnostic tool rather than a therapeutic intervention is researched, this is too rash to state. Please remove this from the Conclusion and Abstract.

#### Minor Comments

##### Overall

7. The authors provide results with IQR; however, no ranges are given. Please describe results as mean (SD) when data are normally distributed or median (25th percentile – 75th percentile) when data are not normally distributed.

8. Formatting of the full manuscript needs some attention. For example, in the Abstract, not all sentences start with a capital letter. Also, it is common in the English language to write number in full up to 20.

9. Please follow the author guidelines of the journal for reporting values and the structure of the manuscript.

##### Title Page

10. The authors state that a clinical trial registration was done. However, it seems that they refer to a registration by a medical ethical review board. Please provide a clinical trial registration or if not applicable, remove it from the title page.

##### Introduction

11. In the first sentence, please state the full name of “emergency department” before using the abbreviation ED.

##### Methods

12. Figure 1 should be formatted. The most common formatting is according to the CONSORT flow diagram.

##### Results

13. Please do not discuss the results in the Results section.

##### Discussion

14. Please end the Discussion section with the strengths and limitations. The secondary findings should be above the Strengths and Limitations section.

## Round 2 Review

I would like to compliment the authors of their extensive changes to the manuscript. I have some minor comments.

### Minor Comments

1. I would suggest changing the sentence “However, there is still no strong evidence that the diagnostic accuracy of POCUS translates into a clinically relevant difference in patient outcomes” in the Introduction, because you also do not provide strong evidence (I do not know if we ever could provide strong evidence). I would suggest that you focus it more on the fact that the impact of using POCUS in the management of patients in the ED is still relatively unknown.

2. I would suggest to start the Discussion section with a short summary of the key findings.

## Abbreviations

CONSORT: Consolidated Standards of Reporting Trials
ED: emergency department
POCUS: point-of-care ultrasound
